# Large-Scale Plasma Peptidomic Profiling Reveals a Novel, Nontoxic, *Crassostrea hongkongensis*-Derived Antimicrobial Peptide against Foodborne Pathogens

**DOI:** 10.3390/md19080420

**Published:** 2021-07-26

**Authors:** Fan Mao, Yongbo Bao, Nai-Kei Wong, Minwei Huang, Kunna Liu, Xiangyu Zhang, Zhuo Yang, Wenjie Yi, Xiao Shu, Zhiming Xiang, Ziniu Yu, Yang Zhang

**Affiliations:** 1 CAS Key Laboratory of Tropical Marine Bio-Resources and Ecology and Guangdong Provincial Key Laboratory of Applied Marine Biology, South China Sea Institute of Oceanology, Innovation Academy of South China Sea Ecology and Environmental Engineering, Chinese Academy of Sciences, Guangzhou 510301, China; maofan@scsio.ac.cn (F.M.); wongnk@stu.edu.cn (N.-K.W.); mwhuang@scsio.ac.cn (M.H.); liukunna18@mails.ucas.ac.cn (K.L.); zhangxiangyu17@mails.ucas.ac.cn (X.Z.); yangzhuo19@mails.ucas.ac.cn (Z.Y.); yiwenjie20@mails.ucas.ac.cn (W.Y.); xiaoshu@scsio.ac.cn (X.S.); zhimingxiang@scsio.ac.cn (Z.X.); 2 Southern Marine Science and Engineering Guangdong Laboratory (Guangzhou), Guangzhou 510301, China; 3 Zhejiang Key Laboratory of Aquatic Germplasm Resources, College of Biological and Environmental Sciences, Zhejiang Wanli University, Ningbo 315100, China; baoyongbo@zwu.edu.cn; 4 Department of Pharmacology, Medical College, Shantou University, Shantou 515063, China

**Keywords:** oyster, plasma, peptidome, antimicrobial peptides, cytotoxicity

## Abstract

Antimicrobial peptides are a fundamental component of mollusks’ defense systems, though they remain a thinly investigated subject. Here, infection by *Vibrio parahemolyticus* triggered a significant increase in antimicrobial activity in oyster plasma. By using PBS-challenged oysters as a control, plasma peptides from immunologically challenged oysters were subjected to peptidomic profiling and in silico data mining to identify bioactive peptides. Thirty-five identified plasma peptides were up-regulated post infection, among which, six up-regulated peptides (URPs) showed a relatively high positive charge. URP20 was validated with significant antibacterial activity. Virtually, URP20 triggered aggregation of bacterial cells, accompanied by their membrane permeabilization. Interestingly, URP20 was found to be active against Gram-positive and Gram-negative foodborne pathogens as well as *Candida albicans*, with no cytotoxicity to mammalian cells and mice. Our study provides the first large-scale plasma peptidomic dataset that identifies novel bioactive peptides in marine mollusks. Further exploration of peptide diversity in marine invertebrates should prove a fruitful pursuit for designing novel AMPs with broad applications.

## 1. Introduction

Overconsumption of antibiotics has led to the rapid emergence and dissemination of antimicrobial resistance in multidrug-resistant pathogens against virtually all classes of existing antimicrobials [1], which threatens to jeopardize the sustainable development of clinical medicine, animal husbandry, aquaculture, and the food industry [2,3]. Meanwhile, rising public needs for safe, fresh, minimally processed, and naturally sourced foods have posed challenges on food security worldwide, and urged research on innovative antimicrobials [4]. Antimicrobial peptides (AMPs) are key effector molecules in host innate defenses in both vertebrates and invertebrates; cationic AMPs possess broad-spectrum activities against microorganisms [5] and have been increasingly recognized as templates for developing alternative antimicrobials to combat multidrug-resistant superbugs [6]. An extensive body of literature has been devoted to investigating AMPs’ structures and modes of action [7], with an emerging focus on potential antimicrobial constituents of food products [8]. 

To date, over 3200 AMPs from various kingdoms (11% from plants, 11% from bacteria, 0.6% from fungi, 74% from animals), along with a more modest inventory of synthetic peptides, have been described. Among those from natural sources, only 2% of the peptides were identified from mollusks [9]. Mollusks are the second largest phylum of invertebrate animals, comprising 23% of all named marine organisms. These sessile marine organisms lack adaptive immunity, and instead depend heavily on the innate immune system including cell-mediated and humoral components for recognition and elimination of invading microbes [10]. Antimicrobial peptides constitute one of the most important components of the innate immunity in mollusks that provides protection against pathogenic microorganisms [11]. While mollusks are increasingly being appreciated as a rich, accessible source of AMPs, only AMPs from mussels have been studied in detail, which include mussel defensins, mytilins, myticins, and mytimycin [12,13,14,15,16]. Moreover, previous works have focused on the purification and characterization of defensins in other mollusk species, such as oyster defensin from gill extracts of the American oyster (*Crassostrea virginica*) [17], the Pacific oyster (*Crassostrea gigas*) [18], and two defensins from *H. discus discus* [19], whereas the pharmaceutical potential of AMPs from marine mollusks remains scarcely explored. Given that innate immune defenses via AMPs prove sufficient to contain microbial infections in mollusks, it seems logical that at least some of such immune components could act as efficient and potent inhibitors of microbial growth [20]. In applied contexts, despite that mollusk AMPs have been characterized in limited species, some have shown great potential as antiviral agents [21]. 

The Hong Kong oyster (*Crassostrea hongkongensis*) is a commercially and nutritionally valuable mollusk species in aquaculture, and is endemic to coasts of the South China Sea. As filter feeders dwelling in intertidal zones, oysters are prone to infections. The haemolymph is a circulating body fluid found in invertebrates, which serves as an immune tissue functionally analogous to the blood of vertebrates [22]. It is primarily composed of haemolymph cells (hemocytes) and the haemolymph plasma. During infections, a variety of specialized proteins are secreted via exocytosis from hemocytes into the plasma, for mounting humoral responses [23]. In our study, we observed that activated plasma from haemolymph following bacterial challenge exhibited great inhibitory activity against *Vibrio parahaemolyticus* growth, suggesting the existence of abundant antimicrobial components in the plasma, presumably including endogenous peptides with potential antimicrobial activity. Thus far, only a very small number of plasma-derived AMPs from marine mollusks have been studied [9]. In addition, due to the complexity of the origins and composition of plasma components in mollusks, it has been relatively difficult to directly purify and identify any endogenous peptides from the plasma. Therefore, it is highly desirable to develop a novel methodological platform for distinguishing plasma peptides from interfering species that occur in high concentration, such as proteins, lipids, and salts, in the marine mollusk *C. hongkongensis*. 

Here, we utilized a mass spectrometry approach as a fundamental tool for profiling and analyzing plasma peptidome in conjunction with peptide isolation and enrichment to improve the workflow of detecting endogenous peptides from *C. hongkongensis* plasma. Candidates of plasma peptidome based on extracts from the bacterially challenged control groups were identified and subsequently subjected to antimicrobial susceptibility assays to further validate potential AMPs in the plasma. We also assessed the cytotoxicity of identified plasma peptides to mammalian cells and in laboratory mice. Thus, we hypothesized that it would be possible to develop potential AMPs from oyster plasma, and provided evidence supporting its applications as potentially relevant to anti-infective treatment, food preservatives, cosmetics, and agricultural uses.

## 2. Results

### 2.1. Large-Scale Peptidome Applied to Oyster Plasma

**Figure 1:**
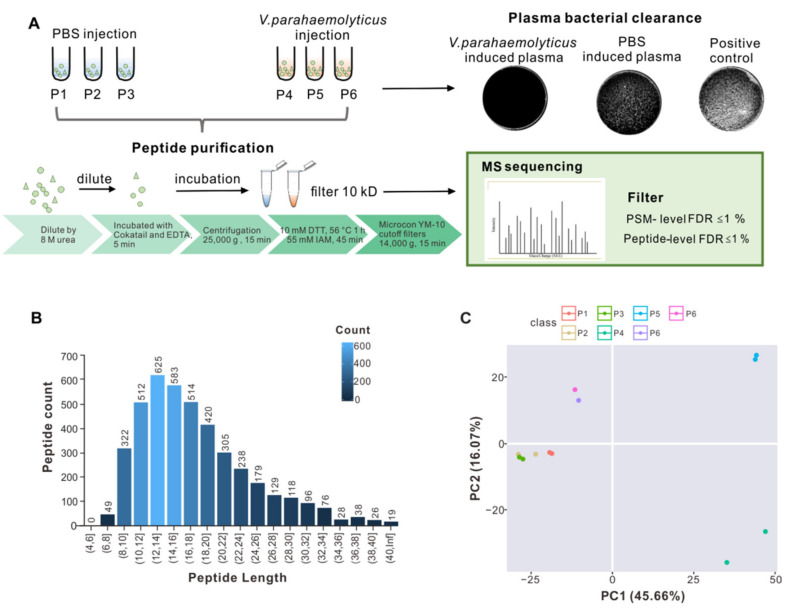
Collection of oyster plasmas for isolation and identification of bactericidal peptides. (**A**) Oysters were treated with *V. parahaemolyticus* or PBS by injection or for 24 h, followed by plasma collection. Bactericidal effects were assessed by incubating (*V. parahaemolyticus* versus PBS-treated) plasmas and LB (positive control) with *V. parahaemolyticus*, whose colony-forming units (CFUs) were subsequently visualized and enumerated in LB agar plates. Peptides of interest were purified from the plasma via indicated steps and subjected to analysis by LC-MS/MS. (**B**) Lengths of the identified peptides in each sample. The names of the samples are shown on the horizontal axis, and the number of polypeptides on the vertical axis. (**C**) Principal component analysis (PCA) score plot of the plasma samples. Colors mark for different samples.

Oyster plasma contains various soluble factors secreted by hemocytes and other cells, which were separated from the haemolymph and collected for antibacterial activity assays. Plasma was collected after *V. parahaemolyticus* injection, with PBS injection being set as a control. Plasma of the *V. parahaemolyticus* challenged group was markedly more bactericidal than that of the PBS-treated control, as shown in Figure 1A (*upper right panel*), suggesting that bacterial challenge rapidly stimulated the expression of bactericidal factors, while the positive control (incubation with LB agar liquid medium for 2 h) contained more *V. parahaemolyticus* cells.

In addition to reported plasma proteins such as lectin [24], other novel antibacterial effector proteins may exist. Peptidomics was herein employed to identify new bioactive peptides with antibacterial activity from plasma based on liquid chromatography and tandem mass spectrometry. To apply large-scale peptidomics to oyster plasma proteins, oyster plasma was extracted from the *V. parahaemolyticus* challenged group (P4, P5, P6) and PBS-treated control (P1, P2, P3). Figure 1A summarized the workflow of the technical processes, which includes peptide purification, peptide filtering, and MS sequencing. To exclude larger protein fragments, these were removed by molecular-weight cutoff (10 kD) spin filters, before MS sequencing of the resultant peptide samples was performed. Peptides were identified by using the MaxQuant integrated Andromeda engine, with filtering at PM-level FDR ≤ 1%, and further filtering at peptide-level FDR ≤ 1% to obtain significant identification results.

### 2.2. Peptide Identification and Informatics Analysis

Each sample in the bacteria-challenged group and PBS-treated control was divided into two sub-samples, respectively, for performing technical repetition. The total spectral count ranged around 617,210 in all the samples, and 39,000 were identified (Table S1). As this study focused mainly on peptides, large proteins (>10 kD) were filtered out manually. Most of the fragments had peptide lengths of 8 to 34 amino acids. Only a few fragments were shorter than 8 amino acids or longer than 34 amino acids (Figure 1B). Then, principal component analysis (PCA) was performed on the subset of identified peptide in each sample, showing that the samples in control group (P1, P2, and P3) formed together and the samples in infection group (P4, P5, and P6) formed in distinct groups, illustrating the individual difference post infection.

### 2.3. Comparative Analysis on Peptide Expression and General Function

**Figure 2:**
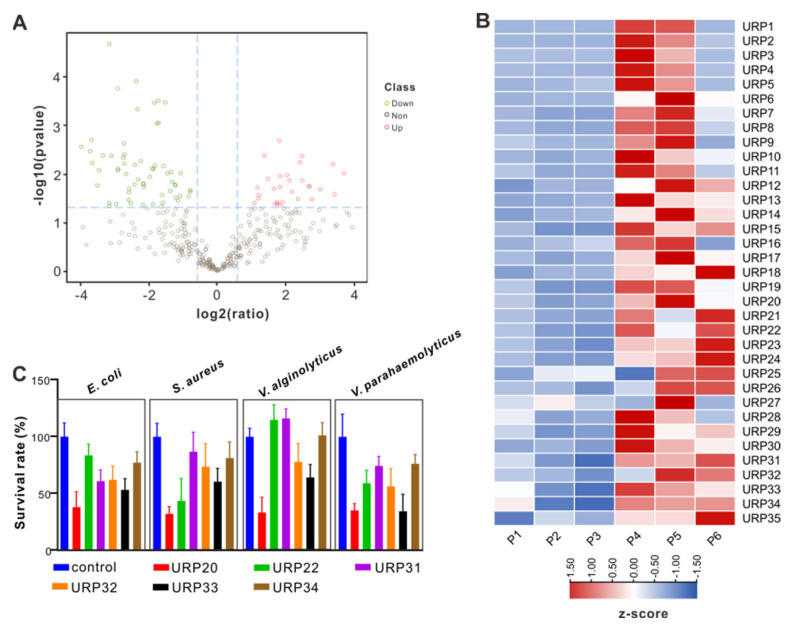
Comparative analysis of the identified peptides. (**A**) Volcano plots show the relationship between fold-changes and significance for vibrio infection group vs control group. The y-axis shows the −log10 (*p*-values) and the x-axis shows the difference in expression as measured in log2 (fold change). (**B**) Heatmaps of gene expression levels of the differentially expressed peptides (DEPs). Peptide expression levels were normalized by z-score normalization method. (**C**) Bactericidal effects of potential antimicrobial peptides (net charge ≥ 2 in up-regulated peptides) at 5 μM on the growth of *E. coli*, *V. alginolyticus*, *V. parahaemolyticus*, and *S. aureus*. Bacterial cells were treated with an equal amount of PBS as a control group. Bactericidal effects were assessed by counting bacterial CFUs on LB agar plates, which were then expressed as percent survival rate relative to that of the control group. Data are presented as means ± SD.

The resulting peptidomes were analyzed, which shows that fewer peptides in the PBS-treated plasma were identified than in the case of bacteria-challenged plasma (Figure S1). A considerable fraction of the peptides was derived from intracellular proteins likely arising from tissue damage under bacterial infection, and was thus not assumed to constitute bioactive peptide. Then, comparative analysis on peptide expression following *V. alginolyticus* infection and PBS infection was performed to elucidate positive active peptides during infection. Differentially expressed peptides (DEPs) were analyzed by comparing peptide abundance in the *V. alginolyticus* infection group with the PBS infection group. In Figure 2A, we use a volcano plot to summarize the magnitude, significance and variability in *V. alginolyticus* infection group. Thirty-five identified peptides indicate an ascending trend in peptide expression, whereas 67 peptides were down-regulated during *V. alginolyticus* infection. Significant protein expression in this study was defined as a *p* value of less than 0.05, with fold changes greater than 1.4 and below 0.7. To illustrate this, the peptide expression level of 35 up-regulated peptides (URPs) is exhibited in Figure 2B.

To facilitate screening of up-regulated peptides (URPs) with antimicrobial potential, the URPs were submitted to the HeliQuest website for prediction on general physico-chemical features, in terms of hydrophobicity, hydrophobic moment, and net charge (z) (Table S2). Consequently, a total of six up-regulated peptides (URP20, URP22, URP31, URP32, URP33, and URP34) were selected for chemical synthesis, since they possessed a positive charge ≥ +2. Then, synthetic peptides were applied for antibacterial activity assay to evaluate their biological activities. We found that URP20 exhibited remarkable inhibitory activity on all bacterial cells tested, in comparison with five other up-regulated peptides (Figure 2C). Furthermore, URP20 showed a medium degree of hydrophobicity (0.25667), compared to peptides with higher hydrophobicity (URP14, 0.601; URP15, 0.629) or lower hydrophobicity (URP17, −0.134), as shown in Table S2. In addition, URP20 has a net charge of +3, which may favor its binding to cell membranes. 

### 2.4. URP20 Showed Broad-Spectrum Antimicrobial Activity against Bacteria and Fungi

**Figure 3:**
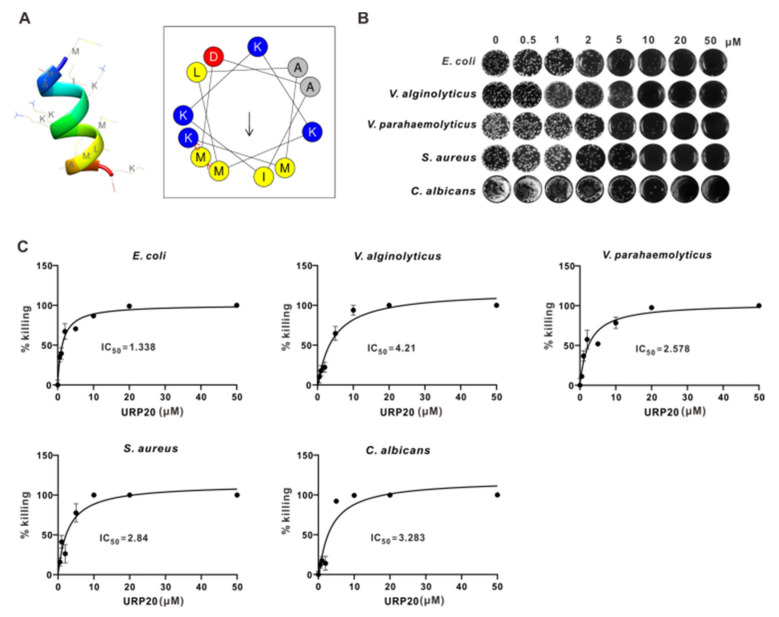
Antimicrobial activity of URP20. (**A**) Peptide structure and helical wheel projection of URP20. Peptide structure was predicted by the PEP-FOLD server. Amino acids are indicated by abbreviations. The peptide helical wheel was predicated on the HeliQuest website. The amino acid composition is indicated by one-letter symbols, namely, Lys (K), Met (M), Leu (L), Ile (I), Ala (A), and Asp (D). Group coloring key: yellow and grey represent nonpolar and hydrophobic amino acids; blue represents basic and charged amino acid; red represents acidic amino acids. The amino acids in red color mark the N- and C-termini of the peptide. An arrow represents the hydrophobic moment. (**B**) Antimicrobial activity of URP20 at various concentrations against Gram-negative bacteria (*E. coli*, *V. parahaemolyticus*, and *V. alginolyticus*), Gram-positive bacteria (*S. aureus*), and fungi (*C. albicans*). (**C**) MIC and MBC were calculated based on statistical data on URP20-dependent killing efficiency in the microbial samples. Data are expressed as a percentage of killing (mean ± SD) relative to that of the control group. IC_50_ values are also indicated. Tests were performed in triplicates.

To establish the antimicrobial mode of action of URP20, MICs and MBCs were analyzed, along with its peptide structure and helical wheel projection. The peptide structure was predicted on the PEP-FOLD server, which confirmed the formation of α-helix. As shown in Table S2, URP20 is a positively charged peptide. The helical wheel projection of URP20 (Figure 3A) indicates where the positively charged amino acids, namely lysines (in blue), are localized. Hydrophobic residues (grey and yellow) are situated on different sides of the wheel, giving rise to a hydrophobic moment (arrow) in the URP20 molecule. Based on this, it is anticipated that URP20 could embed itself in phospholipid membranes with its positive charges pointing outside and its hydrophobic elements facing the hydrophobic core of the membrane.

Antimicrobial susceptibilities of *E. coli*, *V. alginolyticus*, *V. parahaemolyticus*, *S. aureus*, and *C. albicans* toward URP20 were tested. The results (Figure 3B,C) show that URP20 exerted obvious but differential inhibitory effects on the growth of several bacteria. At low concentrations (0.5 μM), URP20 was not inhibitory to bacterial growth. Minimum inhibitory concentrations (MICs) for Gram-negative bacteria (*E. coli*, *V. alginolyticus*, *V. parahaemolyticus*) ranged from 1 to 10 μM (MIC_50_ = 1.338 μM, 4.21 μM, 2.572 μM, respectively), and corresponding minimum bactericidal concentrations (MBCs) ranged from 10 to 20 μM. MICs for Gram-positive bacteria (*S. aureus*) were 1 μM~5 μM (MIC_50_ = 2.84 μM), and corresponding MBCs were 5 μM~10 μM. The MICs for the fungi (*C. albicans*) ranged from 1 μM to 10 μM (MIC_50_ = 3.283 μM), and corresponding MBCs ranged from 10 to 20 μM. According to statistical results (Figure 3C), 0.5 μM of URP20 also had some inhibitory effects on the growth of bacteria. 

### 2.5. URP20 Triggered Aggregation of Bacterial Cells, Accompanied by Microbial Membrane Permeabiliation

**Figure 4:**
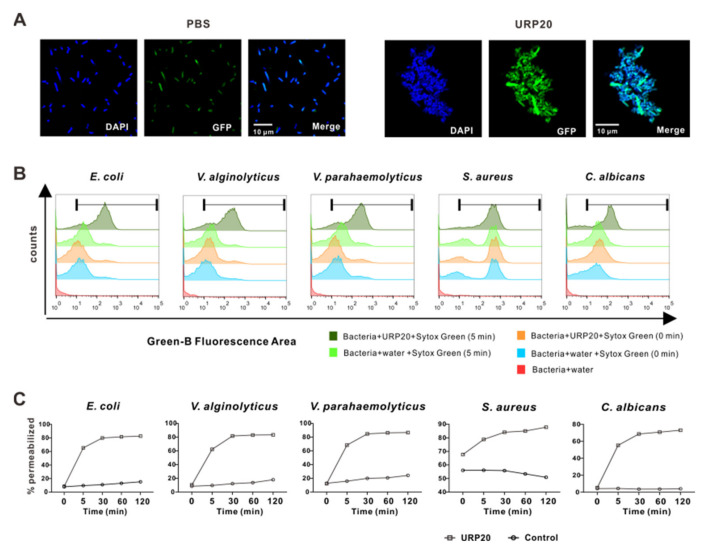
Antimicrobial mechanisms of URP20. (**A**) URP20 triggered aggregation of bacterial cells. Confocal microscopy images were acquired after URP20 challenge (10 min) of bacteria (*E. coli*). DNA was stained with DAPI (blue), and GFP-labelled bacteria emitted green fluorescence. In the control group, URP20 was replaced by an equal amount of PBS. (**B**) Permeabilization efficiency of URP20 after 5 min peptide incubation in bacterial or fungi cells. Membrane permeabilization of bacterial or fungal cells was measured by the Sytox Green assay. Bacterial or fungal cells were exposed to URP20 (20 μM) or an equal amount of water (control). (**C**) Time-lapse study on the effects on URP20 on membrane permeabilization in bacterial or fungal cells. Bacterial or fungal cells were exposed to URP20 (20 μM) or an equal amount of water (control, white circles) at indicated time points (0, 5, 30, 60, and 120 min).

In order to elucidate the antimicrobial mode of action of URP20, we proceeded to observe URP20-treated *E. coli* in confocal fluorescence microscopy (Figure 4A). Bacterial cells were found to adhere to each other in the presence of URP20 (10 μM), while the blank control *E. coli* without peptide treatment showed a dispersed and uniform distribution in the bright field, suggesting that URP20 triggered aggregation of the bacterial cells.

We reasoned that detrimental modifications of the microbial cell surface may occur upon URP20 challenge. We thus focused our subsequent study on the effects of URP20 on microbial membrane permeabilization. SYTOX Green stain is a green-fluorescent nuclear and chromosome counterstain that is impermeant to living cells but can penetrate membranes of dead cells, making it a useful indicator of dead cells within a population. As membrane porosity increases, microbial DNA is stained by internalized SYTOX Green, which accumulates at different levels of fluorescence intensity in flow cytometry. Figure 4B shows the extent of damage to microbial cell membranes by URP20 (at MIC for 5 min). Remarkably, URP20 incubation sharply increased the fluorescence density by 50%, whereas fluorescence density was low in the group without URP20 incubation (5 min) and there was nearly no fluorescence in the group without URP20 incubation and SYTOX Green staining (microbial group added with water only). Then, time-dependent incubation of URP20 and microbial cells were performed (Figure 4C), which shows that membrane permeabilization progressed over time. For example, after URP20 incubation for 30 min, membrane permeabilization of *E. coli*, *V. alginolyticus*, *V. parahaemolyticus*, and *C. albicans* became stable, while that of *S. aureus* occurred more gradually. The results indicate time-dependent damage of URP20 to microbial cells membranes.

### 2.6. URP20 Was Not Cytotoxic to Mammalian Cells or Laboratory Mice

**Figure 5:**
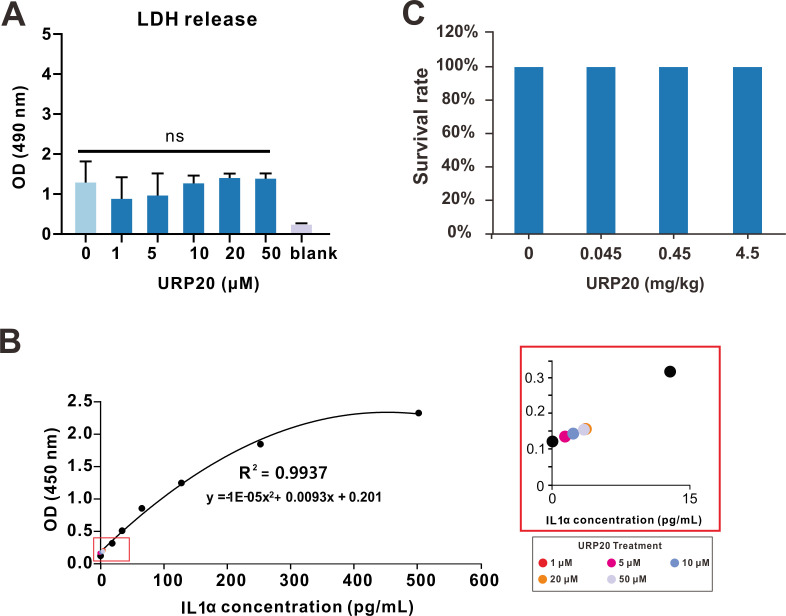
In vitro and in vivo toxicity of URP20. (**A**) HEK293T cells were treated with URP20 in different concentrations (0, 1, 5, 10, 20, 50 μM) for 24 h. Cell death was quantified by measuring lactic dehydrogenase (LDH) release. Data represent means ± SD of optical densities from three independent experiments. Blank: wells with no medium and cells. (**B**) Standard curve of IL1α concentration was established, as displayed in the left panel. Murine J774.1 cells were treated with URP20 at different concentrations (1, 5, 10, 20, 50 μM) for 24 h, followed by detection of IL1α levels by ELISA, as shown in the right panel. Colored dots represent IL1α response corresponding to different URP20 concentrations in treatment. (**C**) C57BL/6 mice were treated with URP20 at indicated concentrations (0, 0.045, 0.45, and 4.5 mg/kg) by intratracheal (i.t.) instillation and subsequently euthanized at 24 h after determination of mobility. Survive rate is displayed above.

Since AMPs may exert toxic effects on host cells, we set out to assess any potential toxicity of URP20 to mammalian cells in vivo or to laboratory mice. In vitro, URP20 elicited negligible LDH release in HEK293T cells (Figure 5A), with minor effects seen in the induction of a proinflammatory response (reflected by IL1α levels) in murine J774.1 cells (Figure 5B) across a broad range of concentrations of up to 50 μM, compared to the control group (no peptides).

To further assess any toxic effects of URP20 in vivo, we tested this in C57BL/6 mice (20 g in weight) by intratracheal (i.t.) instillation of the compound at up to 4.5 mg/kg. The URP20 treatment resulted in no fatalities and appeared to be well tolerated by observation of apparent effects in physical traits such as mobility (Figure 5C), which likewise suggests no toxicity to the host in vivo.

## 3. Discussion

In this study, we presented a novel approach to the extraction of plasma peptides, empowered by mass spectrometric identification and bioinformatics analysis to tease out potential antimicrobial peptides. To our knowledge, ours is the first comprehensive large-scale plasma peptidomic dataset that describes a large number of endogenous peptides from marine mollusk plasma, based on mass spectrometry. Similar peptidomic analysis was performed in Freshwater Mollusk Pomacea poeyana, revealing promising antimicrobial candidates [25]. In this study, low-abundance endogenous peptides were enriched by filtering with an ultrafiltration tube with a 10 kD filter membrane from a highly complex plasma environment with proteins of large heterogeneity in sizes and charges. This threshold was established for the generally accepted size of AMPs [26]. Thereafter, MS detection was performed with an emphasis on in depth peptide identification. Additional functional characterization of endogenous peptides by bioinformatics was employed to decipher their biological meanings. 

Interestingly, plasma from bacteria-challenged oysters showed significantly higher bactericidal activity, implying an increased expression level of antimicrobial components in bacteria-challenged samples. It is clear that some peptides occurred as degradation products from endogenous proteins, due to immunologically induced tissue damage and proteolysis [27]. Previously, it has been demonstrated that some bioactive peptides are well-defined peptides from specific proteolytic degradation of larger proteins [28,29]. For example, histone-derived AMP, known as Abhisin, is an endogenous AMP derived from the *N*-terminal region of histone H2A in disk abalone, with typical antimicrobial peptide characteristics [30]. In addition, another type of endogenous peptides is encoded by DNA coding sequences, such as short reading frames (sORF) [31] and non-coding RNA (ncRNA) [32]. Therefore, it is relatively challenging to develop novel bioactive peptides plainly on the basis of their biological sources. Thus, the analytical strategy and procedural framework used in our study have been proven robust for high-throughput identification of bioactive peptides. 

AMPs are regarded as promising candidates for novel food preservatives in industrial and pharmaceutical applications, due to their relative safety and broad-spectrum antimicrobial activity. Here, we mainly focused on the antimicrobial activity of identified plasma peptides, but not the origin of the peptides, to reduce the peptidomic complexity. Antimicrobial activity of these peptides varied in different bacteria strains, and only URP20-selected plasma peptides showed strong antimicrobial activity. While AMPs are characterized by a wide antimicrobial spectrum, several previous works described specificity of some AMPs toward certain bacterial species. For example, the mussel defensins, MGDs, mytilins, and myticins have shown greater antibacterial activity against Gram-positive than against Gram-negative bacteria [15]. Generally, AMPs target membranes and directly kill microbes by disruptive forces of electrostatic and hydrophobic interactions [33]. Nevertheless, there has been no unifying principles on the actual modes of bacterial killing by AMPs to different species, resulting in limited understanding on the diversity of antimicrobial activity. 

It has been proposed that biophysical determinants of antimicrobial activity include small size, cationicity, and amphipathicity [34]. In this study, URP20 was a plasma peptide identified with potent anti-bacterial and anti-fungal activity. It displayed robust and broad-spectrum antimicrobial activity, which reflected its amino acid composition. Among them, lysine (K), leucine (L), and isoleucine (I) helps to effectively form antimicrobial peptides. The positively charged lysine (K) can bind to negatively charged bacterial components, allowing an AMP to be more tightly attached to a membrane surface. Lysine (K), along with alanine (A), is known for boosting antimicrobial activity [35]. Leucine (L) and isoleucine (I) provide hydrophobic groups to bind bilayers with high affinity, thereby disrupting lipid vesicles and bacterial membranes [36]. Most of the antimicrobial peptides are positively charged alkaline peptides, which interact with anionic substances to increase local osmolality, disrupt cell membranes, and eventually induce cell death [37]. URP20 showed a significant ability to damage to cell membranes of Gram-negative and Gram-positive bacteria and fungi, which suggests a broad spectrum of antimicrobial activity against a microbes. Indeed, major bacterial pathogens that cause foodborne infections include *Staphylococcus aureus*, *Escherichia coli*, *Vibrio cholera,* among others [38]. The robust and broad-spectrum antimicrobial activity of URP20 allowed it to target *Vibrio* spp., *E. coli*, and *S. aureus*, raising the possibility of its use as a potential preservative to extend the shelf-life of food products. Additionally, *Candida albicans* is a fungal species of the human microbiota with the ability to asymptomatically colonize many parts of the body [39]. Although few mollusk AMPs have been tested for antifungal activities, the antimicrobial activity of URP20 against *Candida albicans* seen in this study points to potential of mollusk peptides as lead compounds for developing antifungal agents. In addition, we also showed that, within the range of bactericidal concentrations, URP20 was not cytotoxic or proinflammatory toward mammalian cells and mice, which lends further support to its safe use as naturally occurring antibacterial agents. 

Overall, antimicrobial peptides are a promising new class of naturally sourced components as alternative food preservatives [8,40]. Selective cytotoxicity of cationic AMPs against a broad spectrum of human cancer cells supports their exploration as novel antitumor agents, which may avoid the disadvantages of conventional chemotherapy. Marine invertebrates have been increasingly appreciated as a rich source of novel and AMPs [20]. The development of a large-scale plasma peptidomic strategy has patently facilitated biological characterization and in silico mining of novel marine AMPs. Collectively, we believe that future analysis on peptide diversity in marine invertebrates will inspire new designs of functionally attractive AMPs for biotechnological applications.

## 4. Materials and Methods

### 4.1. Oyster Collection

Hong Kong oysters, *Crassostrea hongkongensis* (two-year old individuals with an average 100 mm shell length), were obtained from oyster culture facilities in Zhanjiang, Guangdong Province, China, and maintained at 22–25 °C in tanks with re-circulating seawater before experiments. The oysters were fed twice a daily with *Tetraselmis suecica* and *Isochrysis galbana*, during acclimation (two weeks) prior to study.

### 4.2. Bacterial Challenge and Plasma Collection

To investigate bactericidal effects of plasma components, 100 oysters were randomly assigned into 2 groups and placed in 2 tanks: the bacterial challenge and control groups. For the experimental group, oysters were challenged by injecting 100 μL *Vibrio parahaemolyticus* (1 × 10^8^ CFU/mL) suspended in phosphate buffer saline (PBS) into the adductor muscle. For the control group, an equal volume of PBS was injected. At 24 h post-challenge, hemolymph was collected from the pericardial cavity through the adductor muscle and immediately centrifuged (700 × *g* for 10 min at 4 °C) to separate the plasma from hemocytes. Every three samples (ten oyster/sample) oysters in one sample were randomly collected in each group after injection (P1, P2, P3 for PBS injection group; P4, P5, P6 for *V. parahaemolyticus* injection group). 

### 4.3. Bacterial Clearance Assay

Twenty microliters of the plasma from individual samples were mixed and incubated with 20 µL *V. parahaemolyticus* (1 × 10^6^ CFU/mL) at 37 °C for 2 h. Equal amounts of PBS and LB agar liquid medium were determined as the negative control and positive control. After two hours of incubation, 10 µL of the mixture was drawn as an inoculum for agar plating. Enumeration of survivors’ colonies (CFU) was performed on LB (Luria-Bertani) agar plates in triplicates.

### 4.4. Peptide Purification

Appropriate amounts of the samples were taken and centrifuged at a high speed of 20,000× *g* for 10 min to remove impurities such as precipitation. The supernatant was placed on ice and diluted with 8 M urea to a final concentration of 10 μg/μL, followed by incubation in the presence of 1 × protease cocktail and 2 mM EDTA for 5 min. Then, DTT (dithiothreitol) was added to a final concentration of 10 mM, followed by incubation for 1 h at 56 °C, and a final concentration of 55 mM IAM (iodoacetamide) was added to the mixture in a darkroom, followed by further incubation for 45 min at room temperature. Next, equal amounts of proteins in each sample were filtered in ultrafiltration tubes with a 10 kD filter membrane and centrifuged in a volume of 400 μL at a time at 14,000 × *g* for 15 min. The resultant filtrates were collected, which were then further purified by a C18 solid-phase extraction column (following a standard protocol involving activation, balance, sample loading, washing, and elution). The eluent was cold-frozen and drained. The drained polypeptides were re-dissolved by 0.1% FA (Formic acid) of an appropriate volume, and their trace amounts were detected by MALDI-TOF mass spectrometry (MS) for quality control, followed by analysis by LC-MS/MS (Thermo Q-Exactive).

### 4.5. MS Sequencing

LC-MS/MS analysis was performed on an UltiMate 3000 UHPLC (Thermo Scientific, Waltham, MA, USA), a prominence nano-HPLC system (Shimadzu, Tokyo, Japan) coupled with Q-Exactive (Thermo Fisher Scientific, Waltham, MA, USA). The peptides were re-dissolved and loaded on trap column (30 μm × 5 mm, μ-Precolumn, Thermo Scientific, Waltham, MA, USA) with buffer A (2% ACN, 0.1% FA) in 5 min, followed by a 55 min gradient: from 5% B (98% ACN, 0.1% FA) to 25% B in 40 min, to 35% B in 5 min, to 80% B in 2 min, to 80% B for 2 min, dropped to 5% within 0.5 min and then kept at 5% B for 5.5 min at a flow rate of 300 nL/min. The sample was then separated and transferred to the mass spectrometry system.

After liquid-phase separation, peptides were ionized by a nanoESI source and then transferred to a Q-Exactive tandem mass spectrometer (Thermo Fisher Scientific, San Jose, CA, USA) for data-dependent acquisition (DDA) mode detection. The main parameters used were: ion source voltage set to 1.6 kV, and the scanning range of primary mass spectrometry to 350~1600 *m/z*; resolution set to 70,000; and an initial *m/z* of secondary mass spectrometry fixed as 100, at a resolution of 17,500. The parent ion screening conditions for secondary fragmentation were: charge, 2+ to 7+; and peak strength of the parent ion, > 10,000 and ranks in the first 20. Ion fragmentation mode was HCD, and fragmentation ions were detected in Orbitrap. The dynamic exclusion time was set to 15 s. The mass spectrometry proteomics data have been deposited to the ProteomeXchange Consortium via the PRIDE partner repository with the dataset identifier PXD025247.

### 4.6. Peptide Identification and Bioinformatics Analysis

The raw MS/MS data were converted to MGF files and searched against the protein sequences from the genome database of *Crassostrea hongkongensis* [41], using MaxQuant 1.5.3.30. The parameters were set as follows: no enzyme selected; fixed modifications of carbamidomethyl (C); variable modification of oxidation (M); acetyl (protein N-term); 4.5 ppm ppm of precursor mass tolerance; 20 ppm of fragment ion tolerances; used match-between-runs (using default parameters); minimal peptide length, 7; and maximal peptide length, 45. The credible peptide identifications were obtained with FDR < 0.01 at both peptide spectrum matches (PSMs) and peptide levels. The identified peptides were applied to gene Ontology (GO) annotation by Blast2GO (version 5.2).

### 4.7. Prediction on Antimicrobial Activity 

Physicochemical properties of up-regulated peptides (URPs) were subjected to analysis by HeliQuest (https://heliquest.ipmc.cnrs.fr/, 2020-07-29), with regard to hydrophobicity, hydrophobic moment, and net charge (z). 

### 4.8. Antimicrobial Susceptibility Assay

Candidate antimicrobial peptides was synthesized by Bankpeptide (Hefei, Anhui, China). For testing antimicrobial activities of the peptides, suspensions (about 10^6^~10^7^ CFU/mL) of *Escherichia coli* DH5α, *Vibrio alginolyticus* ZJ51, *Vibrio parahaemolyticus* E151, *Staphylococcus aureus* ATCC 29213, and *Candida albicans* ATCC 96901 were first prepared. The candidate antimicrobial peptides (5 μM) were separately incubated with the microbial suspensions for 1 h. Subsequently, a sample was taken as an inoculum for agar plating. CFUs (colony forming units) were enumerated on LB (for bacteria) and peptone (for C. albicans) agar plates following overnight culture for 16 h at 37 °C. A control group was set up by adding equal amounts of PBS. Antimicrobial susceptibilities were determined as a percentage of survival compared to that of the control group, and expressed as means ± SD. All tests were performed in triplicates and repeat for three times.

### 4.9. Determination of Minimum Inhibitory Concentrations (MICs) and Minimum Bactericidal Concentrations (MBCs) of URP20

For antibacterial and antifungal assays, MICs and MBCs were determined by plating bacterial/fungal cells on LB or peptone agar plates following incubation with different concentrations of URP20 (0, 0.5, 1, 2, 5, 10, 20, and 50 μM). Bacterial or fungal cells harvested from overnight cultures were washed three times to remove all traces of culture media and adjusted to a density of 10^5^~10^6^ CFU/mL in sterile PBS before exposure to URP20. The bacterial or fungal suspensions were then incubated with or without peptides in PBS for 2 h at 37 °C. Subsequently, 10 microliters of the mixture from each sample were serially diluted in 1000 μL PBS, from which a 10 μL inoculum was plated in LB agar plates in a 12 well plate for overnight culture, followed by imaging and bacterial or fungus colony counts. Data were analyzed using GraphPad Prism (8.0) software and expressed as a percentage (means ± SD) of killing compared to that of the control group. All tests were performed in triplicates and repeated for three times.

### 4.10. Confocal Imaging of URP20 Triggered Assembly of the Bacterial Cells

Following overnight culture, bacteria (*E. coli* DH5α) were washed three times to remove all traces of culture medium and adjusted to a density of 10^7^ CFU/mL in sterile PBS before exposure to URP20. *E. coli* DH5α cells were transformed with a pFPV25.1 plasmid, which enabled them to emit green fluorescence (GFP). After 10 min exposure to URP20, the bacterial cells were washed three times, placed in confocal dishes, and fixed for 10 min in cold paraformaldehyde (4%). Then, cells were stained for DNA for 10 min with 0.25 μg/mL DAPI (4, 6-diamidino-2-phenylindole). Finally, images were acquired with a Leica LP8X confocal fluorescence microscope. 

### 4.11. Membrane Permeabilization Assay

Bacterial and fungal cells harvested from overnight cultures were washed and re-suspended at a density of 10^9^ CFU/mL in PBS, before being incubated with 1 μM Sytox green (Invitrogen, Waltham, MA, USA). Cells (100 μL) were then exposed to URP20 (20 μM) for different durations of time (0, 5, 30, 60, and 120 min), followed by washing three times with PBS. Cells were then subjected to flow cytometry to assess the extent of membrane permeabilization. For the untreated control group, PBS was added to replace the URP20, and the corresponding sample was subjected to flow cytometry, after incubation periods of 0, 5, 30, 60, and 120 min. The cells with no Sytox green and peptide were set as a blank control. Fluorescence intensity was measured at indicated time points. Permeabilization efficiency was determined by the percentage of cells emitting fluorescence.

### 4.12. Cytotoxicity Assay

Potential cytotoxicity of URP20 was assessed through measurement of the release of lactate dehydrogenase (LDH) in the HEK293T cells, purchased from Guangzhou Cellcook Biotech Co.,Ltd. (Guangzhou, China). First, the cells were cultured overnight in DMEM (10% FBS, 1% penicillin-streptomycin solution) and then exposed to URP20 (0–50 μM) for another 24 h incubation under serum-free conditions. Then, cell viability was assessed by measuring the release of LDH with a commercial lactate dehydrogenase assay kit (ab102526; Abcam, Cambridge, UK). 

Mouse J774.1 cells, purchased from Guangzhou Cellcook Biotech Co.,Ltd., were cultured overnight in DMEM (10% FBS, 1% penicillin-streptomycin solution) and allowed to attach. Then, cells were incubated with fresh medium, and treated with the peptide at different concentrations (1, 5, 10, 20, 50 μM) for another 24 h. Subsequently, cytokine levels were measured using an IL1α ELISA kit (PI561; Beyotime, Haimen, China).

### 4.13. In Vivo Toxicity in Mice

Animal experiments were carried out at the Guangdong Laboratory Animals Monitoring Institute, approved and reviewed by Institutional Animal Care and Use Committee of the institute. Wide-type C57BL/6J mice with an average weight of 20 g were anesthetized by isoflurane inhalation and instilled intratracheally with different doses (0, 0.045, 0.45, 4.5 mg/kg) of URP20 in PBS (50 μL) and again 6 h later. Control mice were instilled with an equal amount of PBS without peptide. Survival of the mice was monitored over a period of 24 h after secondary instillation. Statistical analyses on the data were carried out by using GraphPad Prism software.

## Data Availability

The mass spectrometry proteomics data have been deposited to the ProteomeXchange Consortium via the PRIDE partner repository with the dataset identifier PXD025247 (https://www.ebi.ac.uk/pride/archive/projects/PXD025247).

## Supplementary Materials

The following are available online at https://www.mdpi.com/article/10.3390/md19080420/s1, Table S1. An overview of the peptide indetification in each sample; Table S2. Physicochemical properties of up-regulated peptides (URPs); Figure S1 Abundances of identified peptides in each sample;
