# Supplementary material for: Large-Scale Plasma Peptidomic Profiling Reveals a Novel, Nontoxic, Crassostrea hongkongensis-Derived Antimicrobial Peptide against Foodborne Pathogens
Source: Mar Drugs. 2021 Jul 26;19(8):420. doi: 10.3390/md19080420 (PMC8399951; doi:10.3390/md19080420)
Supplement: Supplementary file 1 [file marinedrugs-19-00420-s001.zip › marinedrugs-1308871-supplementary.pdf]

Supplementary Information

Large-scale plasma peptidomic profiling reveals a novel, nontoxic, Crassostrea hongkongensis-derived antimicrobial peptide against foodborne pathogens

Fan Mao [1, 2#], Yongbo Bao [3#], Nai-Kei Wong [1,4#], Minwei Huang [1, 2], Kunna Liu [1, 2], Xiangyu Zhang [1, 2], Zhuo Yang [1, 2], Wenjie Yi [1, 2], Xiao Shu [1, 2], Zhiming Xiang [1, 2], Yang Zhang [1, 2 *], Ziniu Yu [1, 2*]

[1] CAS Key Laboratory of Tropical Marine Bio-resources and Ecology and Guangdong Provincial Key Laboratory of Applied Marine Biology, South China Sea Institute of Oceanology, Innovation Academy of South China Sea Ecology and Environmental Engineering, Chinese Academy of Sciences, Guangzhou, China;

[2] Southern Marine Science and Engineering Guangdong Laboratory (Guangzhou), Guangzhou, China;

[3] Zhejiang Key Laboratory of Aquatic Germplasm Resources, College of Biological and Environmental Sciences, Zhejiang Wanli University, Ningbo, China;

[4] Department of Pharmacology, Shantou University Medical College, Shantou, China.

# These authors contributed equally to this work.

* Correspondence:yzhang@scsio.ac.cn (Y. Zhang); carlzyu@scsio.ac.cn (Z. Yu); Tel.: +86 20 8910 2507

Table S1. An overview of the peptide indetification in each sample

Sample	Total_spectra	Identified_spectra	Identified_peptides(By MS/MS)
P1_1	50970	1414	569
P1_2	51524	1331	560
P2_1	51742	2630	943
P2_2	52416	2004	743
p3_1	51826	2406	874
p3_2	51983	2234	823
P4_1	52397	6027	2353
P4_2	49110	4372	1770
P5_1	51974	4806	1858
P5_2	51553	4857	1850
P6_1	50869	3572	1172
p6_2	50846	3388	1112

Each sample was divided into two copies as technical duplicates.

Table S2. Physicochemical properties of up-regulated peptides (URPs).

Entry	Sequence	Hydrophobicity (H)	Hydrophobic moment (μH)	Charge
URP1	RADISHLQDEVNL	0.31909	0.27154	-3
URP2	VDEATYRADISHLQDEVNL	0.16278	0.17122	-4
URP3	FPVDEATYRADISHLQDEVNL	0.24111	0.17949	-4
URP4	QEMYNGIKEIIQAEKNAK	0.09333	0.39073	0
URP5	AEWVSTTGRSIPDNAIR	0.39727	0.50809	0
URP6	LTIGHRDGILGRSL	0.51	0.16236	0
URP7	PIKAGDKLPAVDL	0.36636	0.36294	1
URP8	DECIGKPNGVYEVGCRS	0.27188	0.40384	-1
URP9	SEGQIVPIALRRF	0.38583	0.13305	1
URP10	ATKVAPELLKDVCAEH	0.33687	0.39616	-1
URP11	ATKVAPELLKD	0.25727	0.49259	0
URP12	WLTIGHRDGILGRSL	0.48643	0.07998	1
URP13	ATKVAPELL	n.d	n.d	n.d
URP14	AILQGDHTTHTAIIAC	0.60125	0.17835	-1
URP15	VDPQTICTQL	0.629	0.39004	-1
URP16	AVFNPCGSSYWEAVGHYDPDHHTTEKNPFALA	0.35467	0.22643	-3
URP17	AEAEDKPTVEEDQHEDDPNYRAPAQK	-0.134	0.04038	-7
URP18	AILQGDHTGHSAIIAC	0.56625	0.1836	-1
URP19	AINRTTTPLDAHTLKIS	0.38375	0.34684	1
URP20	MDAIKKKMLAMK	0.25667	0.35541	3
URP21	AVDTPSATTTNPKRDVNINVRMFLV	0.3244	0.20027	1
URP22	SATTTNPKRDVNINVRMFLV	0.3185	0.14878	2

URP23	ASTRNEANVNIYLHL	0.22857	0.06669	0
URP24	STARNEANVNIYLHL	0.22857	0.08483	0
URP25	TIGHRDGVLGRSL	0.21091	0.26331	1
URP26	VILQSDHNHPGSEQIACCIIGRAQA H	0.454	0.03146	-1
URP27	DDSPCWRLNGRCQWTSEPCGQ	0.371	0.13501	-1
URP28	FPVDEATYRADISHLQD	0.35938	0.14399	-2
URP29	VVKEGQDSELETLLQ	0.27571	0.31248	-3
URP30	ATKVAPELLKDVC	0.3375	0.54883	0
URP31	ATTTNPKRDVNINVRMFLV	0.28833	0.23193	2
URP32	PVSKVFARQIF	0.59909	0.39078	2
URP33	TNPKRDVNINVRMFLV	0.34875	0.17942	2
URP34	TPSATTTNPKRDVNINVRMFLV	0.33409	0.16843	2
URP35	IDNFRPVQPL	0.535	0.75045	0

n.d, not determined.

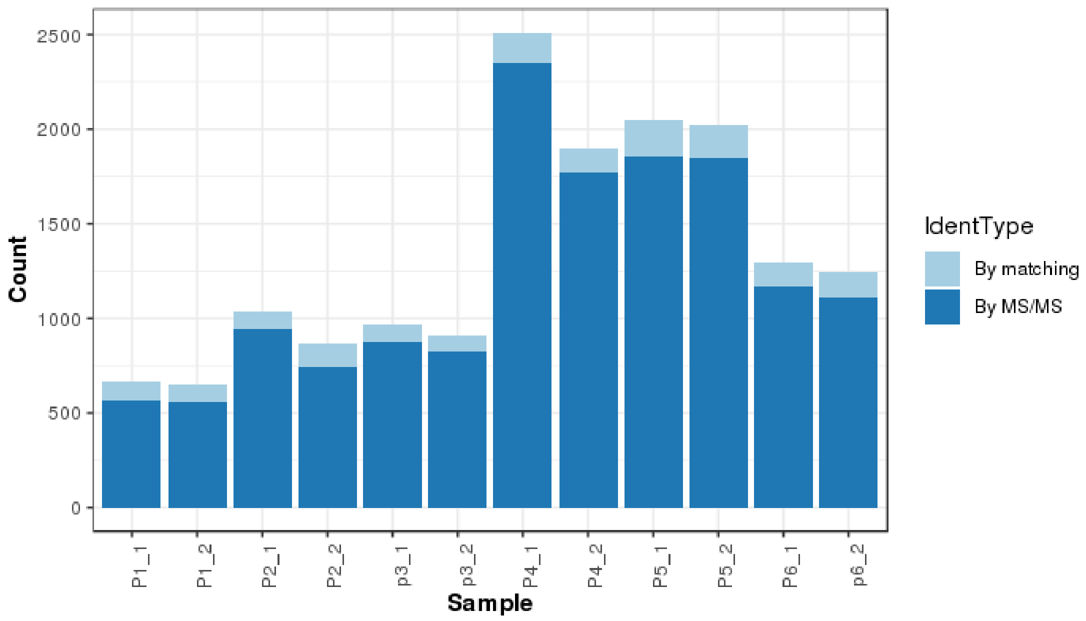

Fig. S1 Abundances of identified peptides in each sample. The horizontal axis shows the length interval of different polypeptides (amino acid residues), and the vertical axis the number of polypeptides in the corresponding interval. “By MS/MS” is the reliable identification result obtained by MaxQuant through secondary spectrum matching. “By matching” is authentication evidence obtained by MaxQuant through the "Match Between runs" algorithm.
